# Risk prediction in medically treated chronic thromboembolic pulmonary hypertension

**DOI:** 10.1186/s12890-021-01495-6

**Published:** 2021-04-20

**Authors:** Ruilin Quan, Yuanhua Yang, Zhenwen Yang, Hongyan Tian, Shengqing Li, Jieyan Shen, Yingqun Ji, Gangcheng Zhang, Caojin Zhang, Guangyi Wang, Yuhao Liu, Zhaozhong Cheng, Zaixin Yu, Zhiyuan Song, Zeqi Zheng, Wei Cui, Yucheng Chen, Shuang Liu, Xiaoxi Chen, Yuling Qian, Changming Xiong, Guangliang Shan, Jianguo He

**Affiliations:** 1 Department of Pulmonary Vascular Disease, State Key Laboratory of Cardiovascular Disease, Fuwai Hospital, National Center for Cardiovascular Disease, Chinese Academy of Medical Sciences and Peking Union Medical College, No. 167, Beilishi Road, Xicheng District, Beijing, 100037 China; 2 Respiratory and Critical Care Medicine, Beijing Institute of Respiratory Medicine, Beijing Chao-Yang Hospital, Capital Medical University, Beijing, 100043 China; 3 Cardiovascular, Tianjin Medical University General Hospital, Tianjin, 300052 China; 4 Peripheral Vascular Department of First Affiliated Hospital, Medical College of Xi’an Jiaotong University, Xi’an, 710061 China; 5 Department of Respiratory Medicine, Xijing Hospital, Fourth Military Medical University, Xi’an, 710032 China; 6 Renji Hospital, Shanghai Jiaotong University School of Medicine, Shanghai, 200240 China; 7 Department of Respiratory, First Affiliated Hospital of Dalian Medical University, Dalian, 116011 China; 8 Department of Cardiology, Wuhan Asia Heart Hospital, Wuhan, 430022 China; 9 Department of Cardiology, Guangdong Provincial People’s Hospital, Guangdong Academy of Medical Sciences, Guangzhou, 510080 China; 10 Chinese PLA General Hospital, Medical School of Chinese PLA, Beijing, 100039 China; 11 Heart Center of Henan Provincial People’s Hospital, Central China Fuwai Hospital, Central China Fuwai Hospital of Zhengzhou University, Zhengzhou, 450003 China; 12 Respiratory Department, The Affiliated Hospital of Qingdao University, Qingdao, 266000 China; 13 Department of Cardiology, Xiangya Hospital, Central South University, Changsha, 410008 China; 14 Department of Cardiology, Southwest Hospital, Third Military Medical University (Army Medical University), Chongqing, 400038 China; 15 Department of Cardiology, The First Affiliated Hospital of Nanchang University, Nanchang, 330006 China; 16 Department of Cardiology, Second Hospital of Hebei Medical University, Shijiazhuang, 050000 China; 17 Department of Cardiology, West China Hospital, Sichuan University, Chengdu, 610041 China; 18 Beijing Anzhen Hospital, Capital Medical University, Beijing, 100029 China; 19 Institute of Basic Medical Sciences, Chinese Academy of Medical Sciences, Beijing, 100037 China

**Keywords:** Chronic thromboembolic pulmonary hypertension, Prognosis, Risk stratification

## Abstract

**Background:**

At present, there is no generally accepted comprehensive prognostic risk prediction model for medically treated chronic thromboembolic pulmonary hypertension (CTEPH) patients.

**Methods:**

Consecutive medically treated CTEPH patients were enrolled in a national multicenter prospective registry study from August 2009 to July 2018. A multivariable Cox proportional hazards model was utilized to derive the prognostic model, and a simplified risk score was created thereafter. Model performance was evaluated in terms of discrimination and calibration, and compared to the Swedish/COMPERA risk stratification method. Internal and external validation were conducted to validate the model performance.

**Results:**

A total of 432 patients were enrolled. During a median follow-up time of 38.73 months (IQR: 20.79, 66.10), 94 patients (21.8%) died. The 1-, 3-, and 5-year survival estimates were 95.5%, 83.7%, and 70.9%, respectively. The final model included the following variables: the Swedish/COMPERA risk stratum (low-, intermediate- or high-risk stratum), pulmonary vascular resistance (PVR, ≤ or > 1600 dyn·s/cm^5^), total bilirubin (TBIL, ≤ or > 38 µmol/L) and chronic kidney disease (CKD, no or yes). Compared with the Swedish/COMPERA risk stratification method alone, both the derived model [C-index: 0.715; net reclassification improvement (NRI): 0.300; integrated discriminatory index (IDI): 0.095] and the risk score (C-index: 0.713; NRI: 0.300; IDI: 0.093) showed improved discriminatory power. The performance was validated in a validation cohort of 84 patients (C-index = 0.707 for the model and 0.721 for the risk score).

**Conclusions:**

A novel risk stratification strategy can serve as a useful tool for determining prognosis and guide management for medically treated CTEPH patients.

*Trial registration*: ClinicalTrials.gov (Identifier: NCT01417338).

**Supplementary Information:**

The online version contains supplementary material available at 10.1186/s12890-021-01495-6.

## Background

Chronic thromboembolic pulmonary hypertension (CTEPH) is characterized by organized thromboembolic obstruction of the pulmonary arteries, which leads to progressively elevated pulmonary vascular resistance, pulmonary hypertension (PH), right heart failure and ultimately death [1]. Pulmonary endarterectomy (PEA) is recommended as the first-line treatment for CTEPH [1, 2]. As reported by the International Registry, PEA can increase the 3-year survival of incident CTEPH patients to 89%, in contrast to the 70% for non-operated patients [3]. However, despite the widely acknowledged benefits of PEA, approximately 40% of CTEPH patients are considered inoperable due to surgical inaccessibility of the thrombi, pulmonary arterial pressure disproportionate with the morphological lesions or the presence of severe comorbidities [4, 5]. For those patients, balloon pulmonary angioplasty (BPA) and riociguat, the only PH-targeted drug for CTEPH approved to date, should be considered alternative treatment options [1, 2]. Meanwhile, for non-operated patients, several studies have ventured to explore predictors of prognosis that can help clinicians identify patients at high risk, determine appropriate treatment strategies, and evaluate the efficacy of the possible treatments [3, 6–13]. As reported, numerous variables, such as World Health Organization (WHO) functional class (FC) [3, 6, 8], 6-min walk distance (6MWD) [6, 8, 13, 14], right atrial pressure (RAP) [6, 13], cardiac index [13], pulmonary vascular resistance (PVR) [12, 14] and brain natriuretic peptide (BNP)/N-terminal pro-BNP (NT-proBNP) [8], have been reported to have prognostic value for CTEPH patients.

However, at present, there is no generally accepted comprehensive prognostic risk prediction model for non-operated CTEPH patients. The 2015 European Society of Cardiology (ESC)/European Respiratory Society (ERS) guidelines proposed a risk stratification strategy for pulmonary arterial hypertension (PAH) with a range of clinical characteristics, biochemical markers and cardiac function and hemodynamics evaluations [2]. This strategy has been further abbreviated and validated in several PAH cohorts [11, 15–17] and two medically treated CTEPH cohorts [10, 11], with all studies demonstrating that patients in the low-risk stratum tend to have better outcomes. Meanwhile, the REVEAL risk score (RRS), another widely used risk assessment tool for PAH, has also had its utility validated for patients with inoperable and persistent/recurrent CTEPH [9]. Nevertheless, the most substantial limitation of the strategies mentioned above is that they are first derived from PAH cohorts without consideration of the prognostic factors specific to CTEPH patients [10, 11]. Furthermore, the inclusion of a broad panel of data, including relevant comorbidities and clinically available biomarkers, would also be helpful in improving the accuracy of risk prediction.

Accordingly, the objectives of the current study were to identify prognostic predictors from a broad range of data, including clinical assessments, comorbid conditions, routinely available biomarkers, evaluations of cardiac/pulmonary function and hemodynamic parameters, in a national prospective multicenter CTEPH registry dataset, and to further establish a risk assessment tool specific to medically treated CTEPH patients.

## Methods

### Study design

In this national multicenter prospective registry study, patients with CTEPH were consecutively recruited from 18 participating medical centers throughout China. We performed retrospective data analysis using the abovementioned prospective CTEPH registry data in the current study. The study protocol was approved by the Institutional Review Board (IRB) of Fuwai Hospital (Approval No. 2009-208), complies with the Declaration of Helsinki, and is registered on ClinicalTrials.gov (Identifier: NCT01417338). Written informed consent was obtained from all enrolled patients.

### Study participants

Patients were enrolled in the registry according to the following criteria: 1) right heart catheterization (RHC) performed within one month before enrollment between August 2009 and July 2018; 2) PH confirmed by RHC with a pulmonary arterial pressure (mPAP) ≥ 25 mmHg and pulmonary arterial wedge pressure (PAWP) ≤ 15 mmHg at rest; 3) CTEPH diagnosed based on mismatch on ventilation/perfusion (V/Q) scintigraphy with at least one large perfusion defect in one segment or two subsegments or evidence of pulmonary vascular lesions on computed tomography (CT)/magnetic resonance imaging/pulmonary angiography; 4) administration of at least 3 months of effective anticoagulation; and 5) age ranging from 14 to 85 years. Patients complicated with systemic vasculitis, severe pulmonary disease such as interstitial fibrosis or other comorbid conditions that could have caused nonthromboembolic pulmonary hypertension or those with a life expectancy of less than half a year were excluded. In the current study, we selected medically treated patients who had not undergone PEA or BPA before enrollment or during follow-up. The validation cohort consisted of medically treated CTEPH patients retrospectively enrolled from four centers across China from October 2006 to July 2009 according to the same criteria above.

### Measurements and data collection

Electrocardiography (ECG), chest X-ray, transthoracic echocardiography, pulmonary function tests, V/Q scintigraphy lung scan, high-resolution CT, pulmonary angiography (if necessary), RHC and laboratory tests were performed to evaluate cardiac and pulmonary function, aid in the diagnosis and guide the treatments of CTEPH. Operability for PEA was assessed by an experienced multidisciplinary team (MDT) in the operation centers. Surgically inoperable CTEPH was defined as CTEPH in which the thrombus was in a peripheral location. For enrolled, medically treated CTEPH patients, the following data were collected: (1) demographics, medical history, clinical symptoms and vital signs; (2) examination results; and (3) treatments.

### Endpoint and follow-up

The primary endpoint of this study was all-cause mortality. Overall survival was measured from the date of RHC to the date of death from any cause. Follow-up was performed by telephone calls, outpatient visits or inpatient admissions every 6 months ± 2 weeks. At each follow-up, vital status was confirmed, as well as surgical events, interventions, and instances of cardiac hospitalization. Patients were followed until death or until the cutoff date of the current study (March 2019).

### Statistical analysis

Continuous variables are presented as the mean ± standard deviation or median [interquartile range (IQR)]. Differences were compared by Student’s t test or the Mann–Whitney U test for two groups and 1-way analysis of variance or the Kruskal–Wallis test for multiple groups, as appropriate. Categorical variables are shown as frequencies and percentages and were compared with the chi-square test. Multiple imputation was used to replace missing values for corresponding variables. Cox proportional hazards analyses were performed to compute hazard ratios (HRs) with 95% confidence intervals (CIs). The proportional hazards assumption was examined by the Schoenfeld residuals method. Univariable Cox analyses were first conducted to screen candidate variables, which were based on the literature and clinical expertise and included demographics, clinical assessments, comorbidities, clinically assessed biomarkers, and variables obtained from pulmonary function tests, echocardiography and RHC. In the univariable analyses, 6MWD, WHO-FC, NT-proBNP, cardiac index, RAP and mixed venous oxygen saturation (SvO_2_), which were included as variables in the Swedish/COMPERA risk stratification method, showed significant prognostic value for mortality [15, 16]. Because the Swedish/COMPERA risk stratification method has previously been validated in CTEPH patients [10], we integrated the six parameters into a composite variable according to the specified rules. Hence, we graded the risks for the six variables from 1 to 3 (1 = low, 2 = intermediate, 3 = high) using the recommended thresholds in the guidelines. The rounded means of these grades were then used to define the risk stratum, which was further included in the multivariable model as a priority. Because categorical specifications are favored in daily practice, other continuous risk factors were dichotomized according to the optimal thresholds determined by maximally selected rank statistics before being entered in the multivariable Cox model [18]. Thereafter, stepwise variable selection with entry and exit criteria (*P* < 0.05) was used to obtain the final model. For routine prognostic assessments, a simplified risk score was then derived by assigning integer numbers to each variable according to the adjusted HRs, and the overall risk score was defined as the sum of the risk points.

Model performance was evaluated for discrimination and calibration. Harrell’s C-index was used to assess the discriminatory power, while the calibration of the 5-year risk prediction was visually evaluated by plotting and comparing the predicted and observed risk. For model comparison, net reclassification improvement (NRI) and integrated discriminatory index (IDI) were used. For NRI, we classified the 5-year mortality into low (< 25%), intermediate (25–50%) and high (> 50%) based on previous literature [11]. Survival was estimated by means of Kaplan–Meier analysis, and difference were compared by the log-rank test. Sensitivity analyses were performed in the following subgroups: (1) newly diagnosed patients, (2) surgically inoperable patients, and (3) a subset that excluded patients with chronic liver disease. Internal validation was evaluated with bootstrapping. External validation was performed to justify whether the derived model and the risk score were also predictive of death in a validation cohort. Differences were considered statistically significant when the two-sided *P* value was < 0.05. All analyses were performed with the R statistical package (version 4.0.0, R Foundation for Statistical Computing, Vienna, Austria).

## Results

### Study cohort

**Fig. 1:**
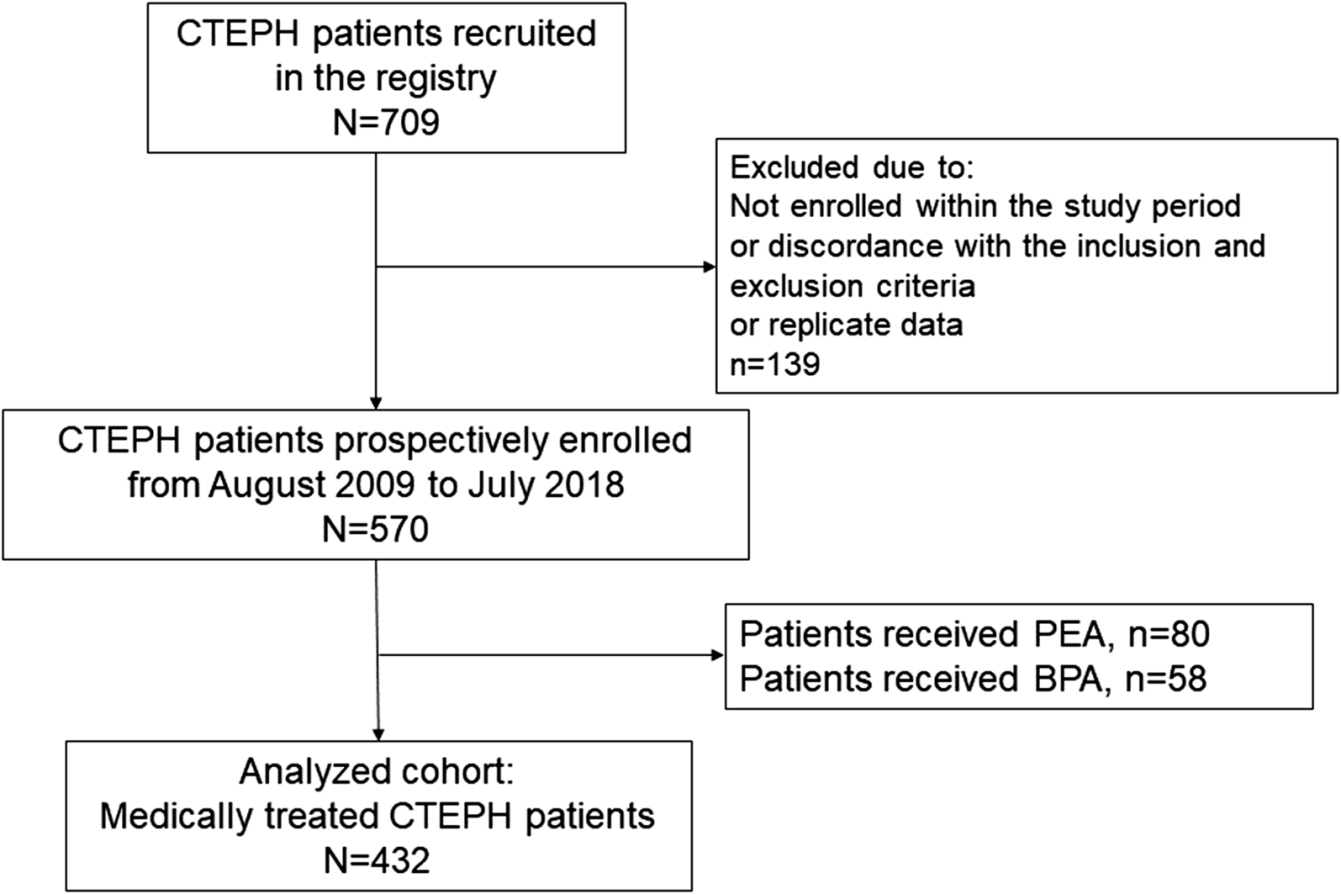
Flowchart of patient selection. CTEPH: chronic thromboembolic pulmonary hypertension; PEA: pulmonary endarterectomy; BPA: balloon pulmonary angiography

A total of 432 medically treated CTEPH patients were enrolled in the current study (Fig. 1). The mean age of the cohort was 53.54 ± 12.25 years, and 52.3% of the patients were males. A total of 59.3% of these patients were newly diagnosed, and 54.2% were surgically inoperable. A total of 251 (58.1%) patients had a history of pulmonary embolism, and chronic kidney disease, defined as an estimated glomerular filtration rate (eGFR) < 60 mL/min/1.73 m^2^, was the most common comorbidity (21.3%). In addition to conventional medical therapies, 55.3% of the patients received at least one PH-targeted drug, while only 9.3% of them received combination therapy. However, as riociguat, the only approved targeted drug for CTEPH, was not available in China before June 2018, it was not in used by any of the patients in the current study cohort. Baseline patient characteristics are presented in Table 1.

**Table 1:** Baseline characteristics of the overall analyzed cohort, survivors and non-survivors

	All N = 432	Survivors N = 338	Non-survivors N = 94	*P* value^#^
Age (years)	53.54 ± 12.25	53.23 ± 12.24	54.68 ± 12.26	0.185
Males, n (%)	226 (52.3)	173 (51.2)	53 (56.4)	0.372
BMI (kg/m^2^)	24.01 ± 3.90	24.15 ± 3.82	23.51 ± 4.15	0.080
Time from symptoms to diagnosis (months)	29.84 ± 33.34	29.43 ± 32.76	31.34 ± 35.50	0.412
Newly diagnosis, n (%)	256 (59.3)	197 (58.3)	59 (62.8)	0.434
WHO-FC, n (%)	0.069			
I/II	207 (47.9)	169 (50.0)	38 (40.4)	
III	199 (46.1)	152 (45.0)	47 (50.0)	
IV	26 (6.0)	17 (5.0)	9 (9.6)	
SBP (mmHg)	116.95 ± 16.78	117.31 ± 16.64	115.68 ± 17.31	0.407
DBP (mmHg)	78.81 ± 40.03	79.21 ± 44.85	77.38 ± 11.11	0.411
6MWD (m)	352.59 ± 107.51	360.93 ± 10.328	322.60 ± 117.28	0.010
Borg dyspnea index	2.76 ± 2.01	2.7 ± 1.98	2.98 ± 2.11	0.386
**Hemodynamics**				
SvO_2_ (%)	63.69 ± 10.59	64.90 ± 9.96	59.35 ± 11.66	< 0.001
HR (beats)	81.67 ± 13.71	81.45 ± 13.91	82.45 ± 13.02	0.573
RVSP (mmHg)	86.80 ± 23.48	85.22 ± 23.64	92.47 ± 22.09	0.012
RVEDP (mmHg)	9.07 ± 7.81	8.48 ± 7.78	11.20 ± 7.56	0.001
RAP (mmHg)	7.18 ± 5.50	6.60 ± 4.94	9.26 ± 6.79	0.001
sPAP (mmHg)	87.22 ± 21.09	85.64 ± 20.71	92.91 ± 21.55	0.006
dPAP (mmHg)	31.53 ± 11.47	30.68 ± 10.65	34.61 ± 13.67	0.007
mPAP (mmHg)	50.36 ± 13.38	49.14 ± 12.50	54.77 ± 15.44	0.002
CI (L·min^−1^·m^−2^)	2.35 ± 0.83	2.47 ± 0.86	1.92 ± 0.57	< 0.001
PAWP (mm Hg)	8.16 ± 3.35	8.17 ± 3.50	8.13 ± 2.78	0.943
PVR (dyn·s·cm^−5^)	1014.38 ± 520.39	919.07 ± 425.89	1357.10 ± 667.84	< 0.001
**Laboratory test**				
NT-proBNP (fmol/L)^*^	810.25 (223.78: 2313.00)	723.00 (181.90: 2093.00)	1458.00 (402.88: 3774.00)	0.003
Hemoglobin (g/L)	149.59 ± 20.38	149.70 ± 20.00	149.23 ± 21.79	0.668
Uric acid (µmol/L)	418.21 ± 126.38	411.47 ± 121.57	442.41 ± 140.40	0.080
Glucose (mmol/L)	5.23 ± 1.25	5.27 ± 1.18	5.09 ± 1.50	0.028
TBIL (µmol/L)	22.44 ± 14.81	21.08 ± 14.00	27.31 ± 29.27	0.001
ALT (IU/L)	29.09 ± 21.39	28.76 ± 21.26	30.27 ± 21.93	0.743
AST (IU/L)	28.65 ± 19.47	27.94 ± 18.54	31.20 ± 22.42	0.020
Creatinine (µmol/L)	82.27 ± 18.95	80.91 ± 18.86	87.15 ± 18.58	0.005
BUN (mmol/L)	6.38 ± 2.00	6.19 ± 1.88	7.06 ± 2.29	< 0.001
**Pulmonary function test**				
FEV1 (% predicted)	80.42 ± 16.88	80.88 ± 16.80	78.77 ± 17.18	0.211
FEV1/FVC (% predicted)	82.08 ± 14.29	82.69 ± 13.82	79.81 ± 15.82	0.055
DLCO (% predicted)	67.37 ± 17.34	67.88 ± 17.46	65.54 ± 16.87	0.148
**Echocardiography**				
LVEF (%)	64.13 ± 7.53	64.07 ± 7.18	64.33 ± 8.72	0.936
LAAPD (mm)	32.68 ± 6.13	32.56 ± 5.95	33.10 ± 6.75	0.746
LVEDD (mm)	37.52 ± 7.11	37.65 ± 11.22	35.83 ± 7.39	0.006
RVAPD (mm)	38.88 ± 11.11	37.65 ± 11.22	43.29 ± 9.52	< 0.001
**Comorbidities, n (%)**				
Atrial fibrillation	17 (3.9)	12 (3.6)	5 (5.3)	0.435
COPD	10 (2.3)	8 (2.4)	2 (2.1)	0.891
Coronary heart disease	31 (7.2)	23 (6.8)	8 (8.5)	0.571
Diabetes	18 (4.2)	15 (4.4)	3 (2.2)	0.593
Hypertension	88 (20.4)	69 (20.4)	19 (20.2)	0.966
Chronic kidney disease^**^	92 (21.3)	61 (18.0)	31 (33.0)	0.002
OSAS	27 (6.3)	26 (7.7)	1 (1.1)	0.019
Thyroid disease	14 (3.2)	9 (2.7)	5 (5.3)	0.198
Pulmonary embolism	251 (58.1)	192 (56.8)	59 (62.8)	0.300
Deep vein thrombosis	104 (24.1)	88 (26.0)	16 (17.0)	0.071
Obesity	47 (10.9)	37 (10.9)	10 (10.6)	0.932
Pericardial effusion	42 (9.7)	30 (8.9)	12 (12.8)	0.260
**Targeted drugs, n (%)**^**※**^				
Any targeted drugs	239 (55.3)	193 (57.1)	46 (48.9)	0.159
ERAs	56 (13.0)	47 (139)	9 (9.6)	0.269
PDE5i	169 (39.1)	132 (39.1)	37 (39.4)	0.915
PCA	37 (8.6)	31 (9.2)	6 (6.4)	0.393
Combination therapy	40 (9.3)	34 (10.1)	6 (6.4)	0.182
**Anticoagulation, n (%)**				
Warfarin	366 (84.7)	279 (82.5)	87 (92.6)	0.017
DOAC	28 (6.5)	27 (8.0)	1 (1.1)	0.016
Heparin	55 (12.7)	41 (12.1)	14 (14.9)	0.592
CCB, n (%)	93 (21.5)	77 (22.8)	16 (17.0)	0.229
Digoxin, n (%)	173 (40.0)	135 (39.9)	38 (40.4)	0.932
Diuretics, n (%)	350 (81.0)	271 (80.2)	79 (84.0)	0.398
Oxygen, n (%)	185 (42.8)	143 (42.3)	42 (44.7)	0.681
MRA, n (%)^┼^	241 (82.8)	191 (81.6)	50 (87.7)	0.274
Statin, n (%)	17 (3.9)	17 (5.0)	0	0.027

BMI: body mass index; WHO-FC: World Health Organization functional class; SBP: systolic blood pressure; DBP: diastolic blood pressure; 6MWD: 6 min walking distance; SvO_2_: mixed venous oxygen saturation; HR: heart rate; RVSP: right ventricular systolic pressure; RVEDP: right ventricular end diastolic pressure; RAP: right atrial pressure; sPAP: systolic pulmonary artery pressure; dPAP: diastolic pulmonary artery pressure; mPAP: mean pulmonary artery pressure; CI: cardiac index; PAWP: pulmonary arterial wedge pressure; PVR: pulmonary vascular resistance; NT-proBNP: N-terminal pro b-type natriuretic peptide; TBIL: total bilirubin; ALT: alanine aminotransferase; AST: aspartate aminotransferase; BUN: blood urea nitrogen; FEV1: forced expiratory volume in 1 s; FVC: forced vital capacity; DLCO: diffusion capacity; LVEF: left ventricular ejection fraction; LAAPD: left atrial anteroposterior diameter; LVEDD: left ventricular end diastolic diameter; RVAPD: right ventricular anteroposterior diameter; COPD: chronic obstructive pulmonary disease; OSAS: obstructive sleep apnea syndrome; ERA: endothelin receptor antagonists; PDE5i: phosphodiesterase-5 inhibitors; PCA: prostacyclin analogues; DOAC: direct oral anticoagulants; CCB: calcium channel blockers; MRA: mineralocorticoid receptor antagonist

^#^comparison between survivors and non-survivors; ^*^median (interquartile range); ^**^CKD stage 3 or more; ^**※**^no patients received stimulator of soluble guanylate cyclase; ^┼^ data only available in 291 patients, among whom 234 were survivors and 57 were non-survivors

During a median follow-up time of 38.73 months (IQR: 20.79, 66.10), 94 patients (21.8%) died. The leading cause of death was right heart failure, and other frequent causes included infection, hemoptysis, and respiratory failure; sudden death was also commonly reported in the cohort. The survival estimates at 1, 3 and 5 years were 95.5% (95% CI: 93.5–97.5%), 83.7% (79.9–87.8%), and 70.9% (65.5–76.7%), respectively.

### Prognostic variables

In univariable Cox analysis, WHO-FC, 6MWD, SvO_2_, RAP, cardiac index, mPAP, PVR, NT-proBNP, total bilirubin (TBIL), creatinine, uric acid, blood urea nitrogen, coronary heart disease and CKD were significant predictors for survival (Additional file 1: Table S1). In addition, the Swedish/COMPERA risk stratum also showed significant predictive value for survival: compared with the low-risk group, the intermediate- (HR 4.008; 95% CI 1.746–9.199) and high-risk (HR 9.740; 95% CI 3.537–26.820) groups demonstrated increased risks for mortality (Additional file 1: Table S1 and Figure S1).

### Risk model and simplified risk score

**Fig. 2:**
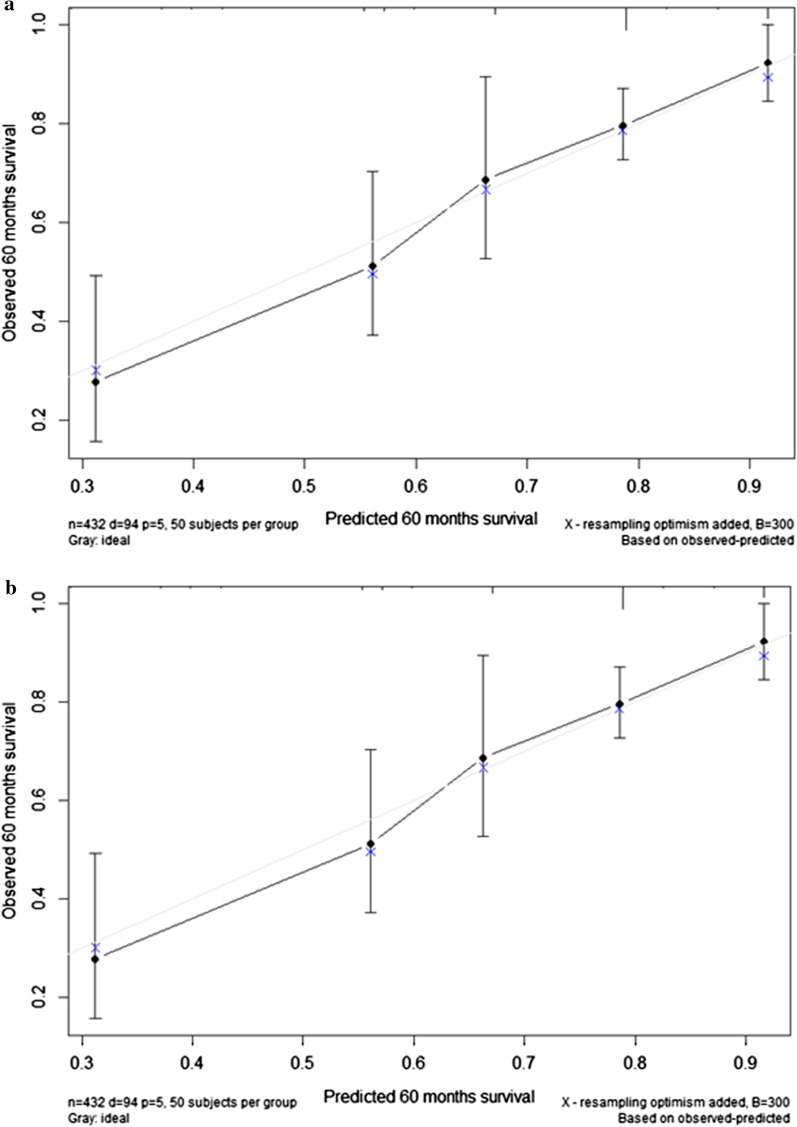
Calibration of the derived model (**a**) and the risk score (**b**). Five-year rates of mortality as predicted versus the observed rates. The diagonal line indicates perfect calibration

According to multivariable Cox proportional hazards analysis, the final model included the following variables: the Swedish/COMPERA risk stratum (low-, intermediate- or high-risk stratum), PVR (≤ or > 1600 dyn s/cm^5^), TBIL (≤ or > 38 µmol/L) and CKD (no or yes). The adjusted HRs and CIs are presented in Table 2. The model showed a C-index of 0.715 (95% CI 0.660–0.770) with good calibration (Fig. 2a).

**Table 2:** Multivariate Cox proportional hazards model for predicting mortality in medically treated CTEPH patients

	Hazard ratio	95% Confidence interval	*P* value	Score
**The Swedish/COMPERA risk stratum**				
Low risk	–	–	–	0
Intermediate risk	2.816	1.206–6.575	0.017	+ 3
High risk	5.889	2.097–16.542	< 0.001	+ 6
**Pulmonary vascular resistance**				
≤ 1600 dyn·s/cm^5^	–	–	–	0
> 1600 dyn·s/cm^5^	2.458	1.562–3.869	< 0.001	+ 3
**Total bilirubin**				
≤ 38 µmol/L	–	–	–	0
> 38 µmol/L	2.288	1.392–3.763	0.001	+ 2
**Chronic kidney disease**				
No	–	–	–	0
Yes	1.671	1.067–2.617	0.025	+ 2

To simplify its use in clinical practice, a risk score was then derived based on the adjusted coefficients of the above model (Table 2). The overall risk score, which ranged from 0 to 13, demonstrated good discriminatory power similar to that of the model, with a C-index of 0.713 (95% CI 0.658–0.768). Figure 2b shows that the risk score was also well calibrated.

Compared with the model consisting only of the Swedish/COMPERA risk stratum, both the full model [NRI 0.300 (0.100–0.542); IDI 0.095 (0.041–0.191)] and the risk score [NRI 0.300 (0.091–0.480); IDI 0.093 (0.044–0.169)] demonstrated improved discriminatory performance (Table 3). Based on the distribution of the risk score, we divided the patients into 3 risk groups, with low-, intermediate- and high-risk groups determined by risk scores of 0–3, 4–5 or ≥ 6 points, respectively.

**Table 3:** C-index, NRI and IDI of the derived model and risk score

	C-index	NRI (95% CI)	*P* value	IDI (95% CI)	*P* value
The Swedish/COMPERA risk stratum	0.616 (0.571–0.661)	–	–	–	–
Model^*^	0.715 (0.660–0.770)	0.300 (0.100–0.542)	0.01	0.095 (0.041–0.191)	< 0.01
Risk score^**^	0.713 (0.658–0.768)	0.300 (0.091–0.480)	< 0.01	0.093 (0.044–0.169)	< 0.01

NRI: net reclassification improvement; IDI: integrated discriminatory index; CI: confidence interval

^*^NRI and IDI compared to the Swedish/COMPERA risk stratum; ^**^NRI and IDI compared to the Swedish/COMPERA risk stratum

**Fig. 3:**
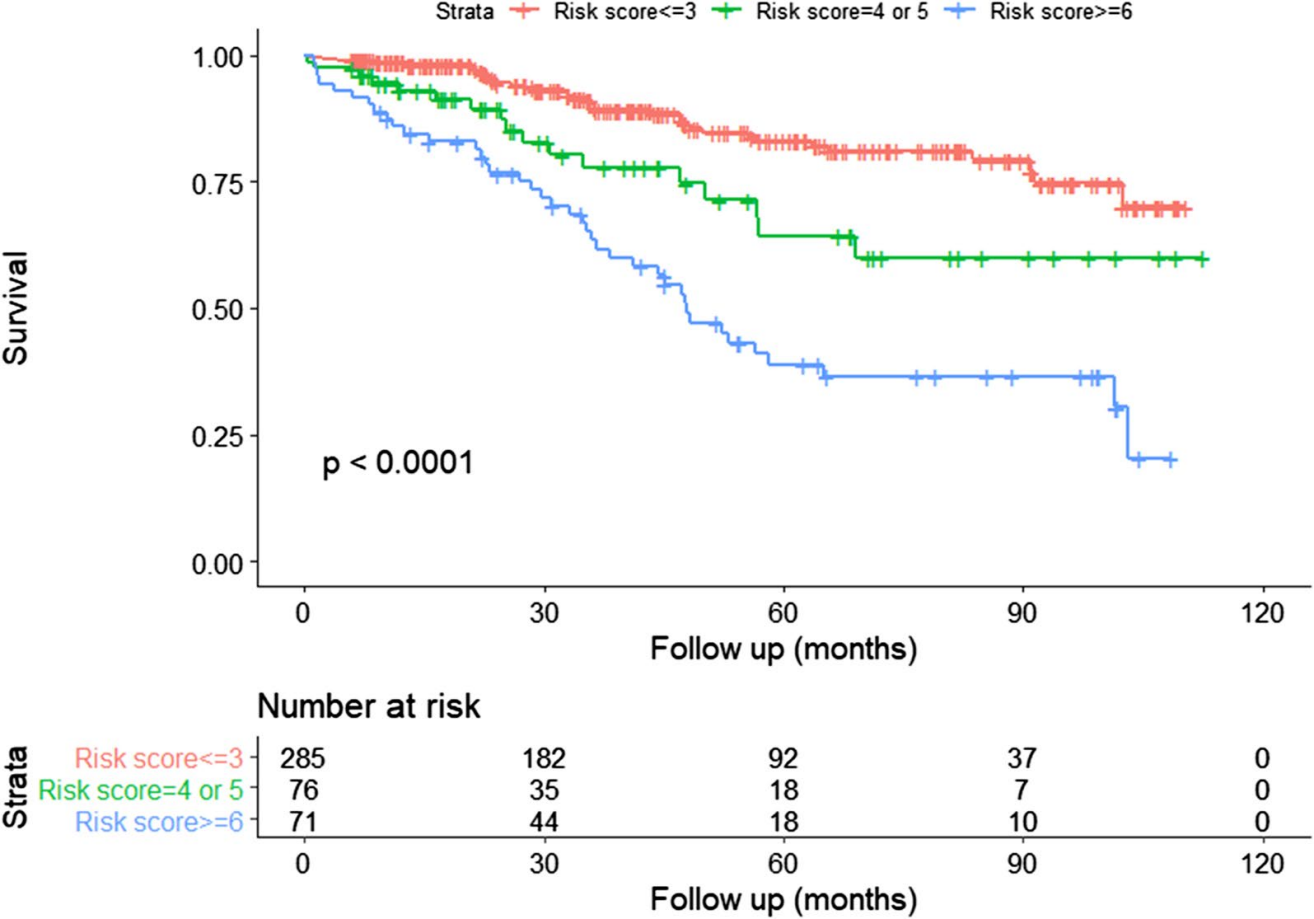
Comparison of Kaplan–Meier survival curves according to the derived risk score (0–3, 4–5 or ≥ 6 points)

The characteristics of the three subgroups are shown in Additional file 1: Table S2. The variables included in the derived risk model differed significantly between the three risk score groups; furthermore, the survival differences between the three risk categories were statistically significant, with *P* < 0.0001 for all comparisons, *P* = 0.006 for the 0–3 versus 4–5 points group comparison, *P* = 0.007 for the 4–5 versus ≥ 6 points group comparison and *P* < 0.001 for the 0–3 versus ≥ 6 points group comparison (Fig. 3). The estimated 5-year survival rates for the three groups were 91.9% (84.9–99.5%), 79.0% (71.9–868%) and 49.3% (40.0–60.7%), respectively (Table 4).

**Table 4:** Estimated 1-, 3- and 5-year survival of the three risk groups

Risk score	Number	Estimated 1-year survival, % (95% CI)	Estimated 3-year survival, % (95% CI)	Estimated 5-year survival, % (95% CI)
0–3	285	98.6 (97.2–1.00)	90.9 (87.2–94.8)	83.1 (77.6–89.0)
4–5	76	93.0 (87.2–99.1)	77.8 (67.0–90.3)	64.3 (50.6–81.7)
≥ 6	71	85.8 (78.1–94.4)	63.4 (52.6–76.4)	38.9 (27.9–54.0)

CI: confidence interval

### Sensitivity analysis and external validation

Sensitivity analyses were performed in three subgroups of patients: patients who were newly diagnosed, those who were surgically inoperable and those without chronic liver disease (excluding 5 patients with chronic hepatitis). The baseline characteristics are shown in Additional file 1: Tables S3-S5. Both the derived model [C-index in newly diagnosed patients: 0.709 (95% CI 0.638–0.780), in surgically inoperable patients: 0.737 (0.649–0.825), in patients without chronic liver disease: 0.718 (0.663–0.773)] and the risk score [C-index: 0.709 (0.640–0.778), 0.743 (0.655–0.831), 0.716 (0.661–0.771), respectively] showed consistent significant predictive value for survival in subgroup analyses. The three risk groups defined by the new risk score also showed significant discriminatory power (Additional file 1: Tables S6-S8 and Figures S2-S4).

The validation cohort consisted of 84 medically treated CTEPH patients with a mean age of 52.01 ± 12.94 years. Twenty-nine patients (34.5%) died during a median follow-up time of 103.56 months (IQR 48.70, 120.00). The survival estimates at 1, 3 and 5 years were 96.4%, 84.2%, and 75.7, respectively. The baseline data of this cohort are presented in Additional file 1: Table S9. Both the derived model [C-index: 0.707 (95% CI: 0.623–0.791)] and the risk score [C-index: 0.721 (0.633–0.809)] demonstrated consistent performance in the validation cohort (Additional file 1: Figure S5). Due to the limited sample size, no subgroup analyses were further performed in the validation cohort.

## Discussion

The present study identified predictors for survival from a broad range of data collected for the study group of medically treated CTEPH patients and thereafter established a new risk assessment tool specific to CTEPH patients. To the best of our knowledge, this is the first risk prediction model specifically derived from the data from CTEPH patients.

Previous studies have reported that numerous variables are associated with the outcomes of medically treated CTEPH patients [3, 6–13]. However, regarding risk prediction tools, published and recommended risk stratification strategies have only been derived for PAH, including the RRS [19, 20], the recommended strategy from the ESC/ERS guidelines [2] and its three abbreviated versions [15–17]. Although these strategies have been validated in CTEPH patients [9–11], it is unknown whether other variables relevant to CTEPH patients would add incremental value. The current study derived a new risk prediction strategy based on real-life registry data from medically treated CTEPH patients and demonstrated that the newly derived prediction model, which combined the Swedish/COMPERA risk stratification method with PVR, serum TBIL and CKD, performed well in predicting survival in those patients. It should be noted that despite the validation of the Swedish/COMPERA risk stratification method, the six variables included in the stratum have also been reported to be individually associated with the outcomes of non-operated CTEPH patients [6, 8, 13, 14]. In addition, PVR is also widely acknowledged as a prognostic factor for CTEPH patients [12, 14].

In contrast to the above-listed variables, TBIL and CKD have been infrequently reported as risk factors in CTEPH patients. In a small sample of 77 inoperable CTEPH patients, the serum concentration of TBIL was found to be an independent prognostic predictor for mortality, with patients whose TBIL ≥ 23.7 µmol/L having markedly worse survival [21]. Similarly, hyperbilirubinemia (serum TBIL > 1.2 mg/dL) was also reported as a predictor of mortality in PAH patients [22]. Furthermore, among biomarkers related to hepatic function, elevated total bilirubin could be the strongest predictor for the adverse outcome of cardiovascular death, superior to transaminases in sensitivity to hemodynamic abnormalities [23]. As chronic liver disease can also affect variables concerning liver function, such as total bilirubin, we further performed sensitivity analyses based on a subgroup consisting of patients without chronic liver disease, where 5 patients with chronic hepatitis were excluded from the cohort. Notably, after excluding these patients, the derived risk model and the risk score showed consistent significant prognostic power. Similar to hepatic dysfunction, right heart failure may also be the potential link between CTEPH and CKD. Renal insufficiency is included in the original RRS [19, 20]; the latest version, RRS 2.0, has updated this category as eGFR < 60 mL/min/1.73 m^2^ or renal insufficiency, as renal function is an important risk predictor for PAH patients [24, 25]. Our study further supports the prognostic use of renal insufficiency in CTEPH patients. Regarding utility, the risk prediction tools derived in our study also emphasize the importance of controlling these comorbid conditions, which could have significant effects on the outcomes.

The 1-, 3- and 5-year survival estimates for medically treated CTEPH patients in the current study cohort (95.5%, 83.7%, 70.9%) were higher than those of the International Registry (1- and 3-year survival estimates: 88% and 70%, respectively)^3^ or those reported by Delcroix et al. (1-, 3-, and 5-year survival estimates: 92.0%, 74.7%, and 59.8%, respectively) [10]. Other differences with this latter study, such as the younger cohort age (53 vs. 69 years), the much lower percentages of comorbidities (any comorbidities: 70% versus 91%), and of patients with intermediate risk (18% versus 68%) and the smaller number of patients who received PH-targeted therapy–especially the unavailability of stimulators of soluble guanylate cyclase (sGCs) in our cohort (versus 37% use in Delcroix et al.)–should be elucidated [10]. The potential reason for these differences may be the disparity in the enrolled patients, as the current registry also enrolled previously diagnosed patients, which could lead to potential survival bias. Therefore, the survival estimates are more similar to those of Spanish Registry of Pulmonary Arterial Hypertension (REHAP) registry (1-, 3-, and 5-year survival estimates: 92.6%, 80.7%, and 64.9%, respectively), which included both newly and previously diagnosed patients [26, 27]. As the cohort in the current study included both newly and previously diagnosed patients, similar to daily practice, our results can be more broadly generalized to any clinical scenario. Furthermore, it should also be noted that we performed sensitivity analysis in the subgroup of newly diagnosed patients, and the model illustrated consistently good performance in risk prediction.

Despite the fact that PEA is recommended as the first-line treatment for CTEPH, only 14% of the patients in our study underwent this procedure, while approximately 46% who were surgically operable did not. The low rate of PEA could be largely attributed to the unbalanced development of medical centers between different areas, as there are only three surgical centers in China, all located in Beijing. Furthermore, the high financial cost of PEA could be another barrier for patients to undergo the procedure. Regarding its effect on the study, as shown in the sensitivity analysis, the model performed consistently well for surgically inoperable patients. Additionally, it should be noted that although the surgery rate was much lower than that in Western countries, it reflects real-world experience regarding the treatment of CTEPH in developing countries to some extent.

We regarded the six variables included in the Swedish/COMPERA method as a composite variable without redefining the existing categories. Due to the relatively small number of events, the significant predictive value of the six parameters as continuous variables in the univariable analyses, and the fact that the risk strata have been previously validated and have shown consistent discriminative power in our cohort [10], we did not include each variable separately with new cutoffs, which may have helped avoid overfitting. However, it is possible that the stratification strategy with the cutoff values first derived for PAH might not be suitable for patients with CTEPH. Therefore, further studies are still needed to investigate appropriate cutoff values specific to CTEPH.

Our study has several limitations. First, we only evaluated variables at baseline to establish and validate the model and did not utilize the data from follow-up visits, as these data were incomplete; only 34 patients (7.9%) had data on follow-up RHC. Among these patients, 2 met the primary endpoint during the follow-up period. Therefore, both the small sample size and the low event rate in the follow-up data prevented us from performing further analyses. However, as reevaluations at follow-ups are necessary for risk assessment and treatment guidance, further studies are required to include information obtained at follow-up to achieve better assessments. Second, as mentioned above, we did not include the six variables in the Swedish/COMPERA risk strata separately and did not find new cutoff values, which could lead to potential inconsistency with the actual categories for the CTEPH patients. Finally, as we only performed external validation in a retrospective cohort with a small sample size, further external validation in independent larger cohorts is required.

## Conclusion

Our novel risk stratification strategy can serve as a useful tool for determining prognosis and guiding management for medically treated CTEPH patients.

## Supplementary Information

**Additional file 1**. **Figure S1**. Comparison of Kaplan-Meier survival curves according to the Swedish/COMPERA risk stratum (Low-, intermediate- and high-risk groups). **Figure S2**. Comparison of Kaplan-Meier survival curves according to the derived risk score (0-3, 4-5 or ≥ 6 points) in newly diagnosed CTEPH patients. **Figure S3**. Comparison of Kaplan-Meier survival curves according to the derived risk score (0-3, 4-5 or ≥ 6 points) in surgically inoperable CTEPH patients. **Figure S4**. Comparison of Kaplan-Meier survival curves according to the derived risk score (0-3, 4-5 or ≥ 6 points) in patients without chronic liver disease. **Figure S5**. Calibration of the derived model (A) and the risk score (B) in the validation cohort. **Table S1**. Univariate Cox proportional hazards analyses of candidate variables for all-cause mortality in the overall analyzed cohort. **Table S2**. Baseline characteristics of the 3 risk groups classified by the new derived risk score. **Table S3**. Baseline characteristics of the newly diagnosed CTEPH patients. **Table S4**. Baseline characteristics of the surgically inoperable CTEPH patients. **Table S5**. Baseline characteristics of patients without chronic liver disease. **Table S6**. Estimated 1-, 3- and 5-year survival of the three risk groups in newly diagnosed CTEPH patients. **Table S7**. Estimated 1-, 3- and 5-year survival of the three risk groups in surgically inoperable patients. **Table S8**. Estimated 1-, 3- and 5-year survival of the three risk groups in patients without chronic liver disease. Table S9. Baseline characteristics of the validation cohort.

## Data Availability

The datasets used and/or analyzed during the current study are available from the corresponding author on reasonable request.
