# Supplementary material for: Risk prediction in medically treated chronic thromboembolic pulmonary hypertension
Source: BMC Pulm Med. 2021 Apr 20;21:128. doi: 10.1186/s12890-021-01495-6 (PMC8056726; doi:10.1186/s12890-021-01495-6)
Supplement: Supplementary file 1 — Additional file 1. Figure S1. Comparison of Kaplan-Meier survival curves according to the Swedish/COMPERA risk stratum (Low-, intermediate- and high-risk groups). Figure S2. Comparison of Kaplan-Meier survival curves according to the derived risk score (0-3, 4-5 or ≥ 6 points) in newly diagnosed CTEPH patients. Figure S3. Comparison of Kaplan-Meier survival curves according to the derived risk score (0-3, 4-5 or ≥ 6 points) in surgically inoperable CTEPH patients. Figure S4. Comparison of Kaplan-Meier survival curves according to the derived risk score (0-3, 4-5 or ≥ 6 points) in patients without chronic liver disease. Figure S5. Calibration of the derived model (A) and the risk score (B) in the validation cohort. Table S1. Univariate Cox proportional hazards analyses of candidate variables for all-cause mortality in the overall analyzed cohort. Table S2. Baseline characteristics of the 3 risk groups classified by the new derived risk score. Table S3. Baseline characteristics of the newly diagnosed CTEPH patients. Table S4. Baseline characteristics of the surgically inoperable CTEPH patients. Table S5. Baseline characteristics of patients without chronic liver disease. Table S6. Estimated 1-, 3- and 5-year survival of the three risk groups in newly diagnosed CTEPH patients. Table S7. Estimated 1-, 3- and 5-year survival of the three risk groups in surgically inoperable patients. Table S8. Estimated 1-, 3- and 5-year survival of the three risk groups in patients without chronic liver disease. Table S9. Baseline characteristics of the validation cohort. [file 12890_2021_1495_MOESM1_ESM.doc]

**Risk Prediction in Medically Treated Chronic Thromboembolic Pulmonary Hypertension**

Ruilin Quan, Yuanhua Yang, Zhenwen Yang, et al.

**Supplementary Appendix**

**Contents**

**Supplementary figures**

Figure S1. Comparison of Kaplan-Meier survival curves according to the Swedish/COMPERA risk stratum (Low-, intermediate- and high-risk groups).

Figure S2. Comparison of Kaplan-Meier survival curves according to the derived risk score (0-3, 4-5 or ≥ 6 points) in newly diagnosed CTEPH patients.

Figure S3. Comparison of Kaplan-Meier survival curves according to the derived risk score (0-3, 4-5 or ≥ 6 points) in surgically inoperable CTEPH patients.

Figure S4. Comparison of Kaplan-Meier survival curves according to the derived risk score (0-3, 4-5 or ≥ 6 points) in patients without chronic liver disease.

Figure S5. Calibration of the derived model (A) and the risk score (B) in the validation cohort.

**Supplementary tables**

Table S1. Univariate Cox proportional hazards analyses of candidate variables for all-cause mortality in the overall analyzed cohort.

Table S2. Baseline characteristics of the 3 risk groups classified by the new derived risk score.

Table S3. Baseline characteristics of the newly diagnosed CTEPH patients.

Table S4. Baseline characteristics of the surgically inoperable CTEPH patients.

Table S5. Baseline characteristics of patients without chronic liver disease.

Table S6. Estimated 1-, 3- and 5-year survival of the three risk groups in newly diagnosed CTEPH patients.

Table S7. Estimated 1-, 3- and 5-year survival of the three risk groups in surgically inoperable patients.

Table S8. Estimated 1-, 3- and 5-year survival of the three risk groups in patients without chronic liver disease.

Table S9. Baseline characteristics of the validation cohort.

**Figure S1. Comparison of Kaplan-Meier survival curves according to the Swedish/COMPERA risk stratum (Low-, intermediate- and high-risk groups).**

**
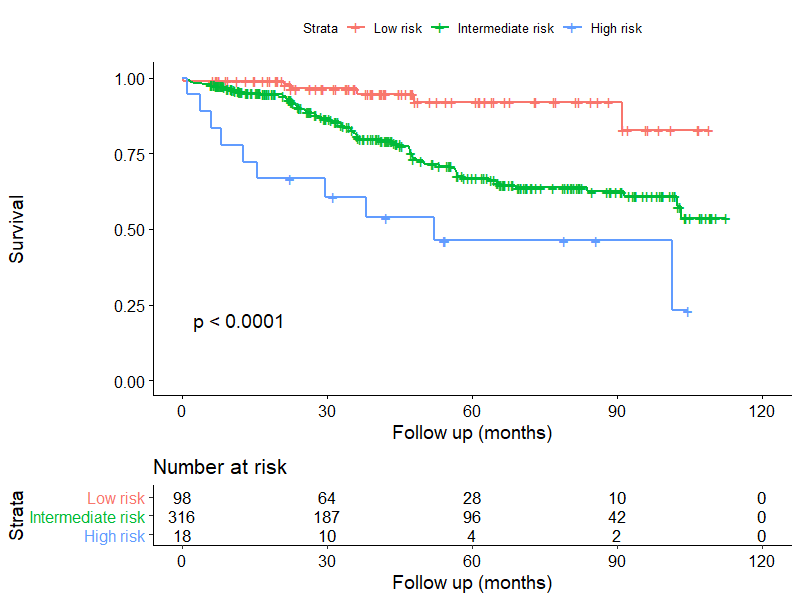
**

**Figure S2. Comparison of Kaplan-Meier survival curves according to the derived risk score (0-3, 4-5 or ≥ 6 points) in newly diagnosed CTEPH patients.**

**
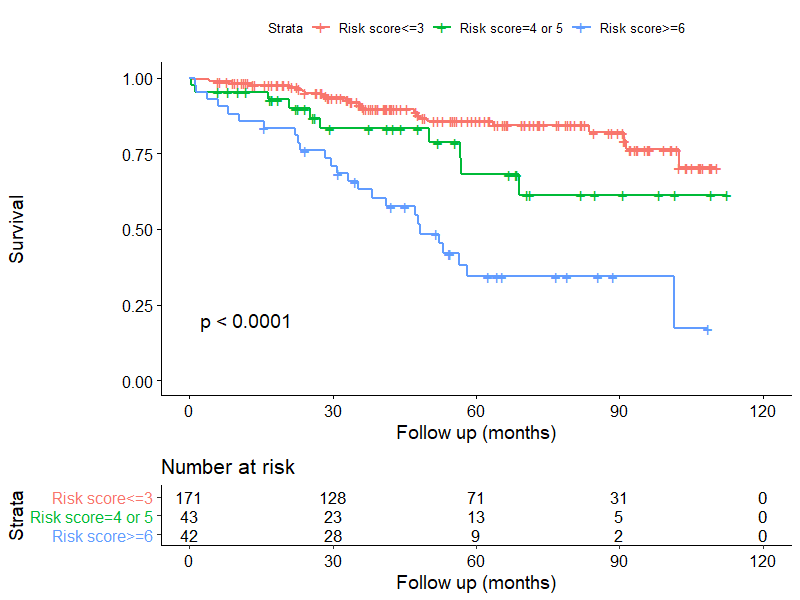
**

**Figure S3. Comparison of Kaplan-Meier survival curves according to the derived risk score (0-3, 4-5 or ≥ 6 points) in surgically inoperable CTEPH patients.**

**
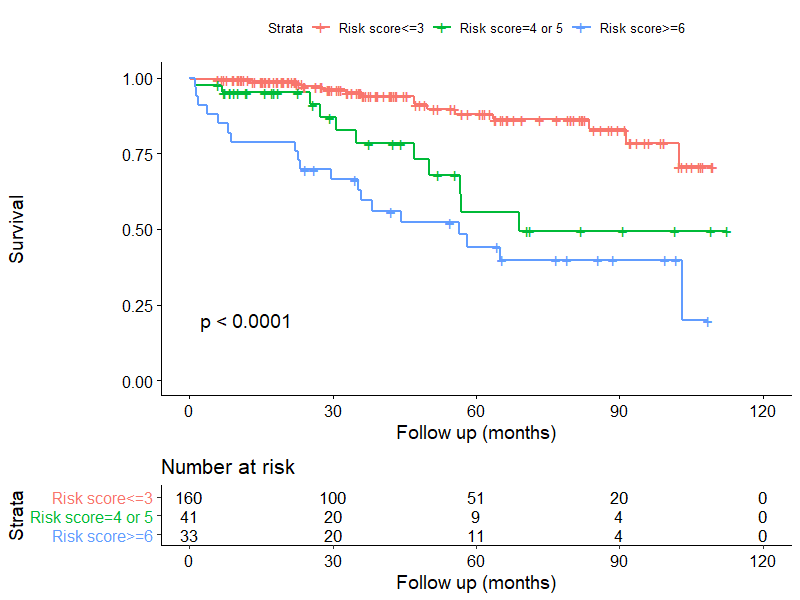
**

**Figure S4. Comparison of Kaplan-Meier survival curves according to the derived risk score (0-3, 4-5 or ≥ 6 points) in patients without chronic liver disease.**

**
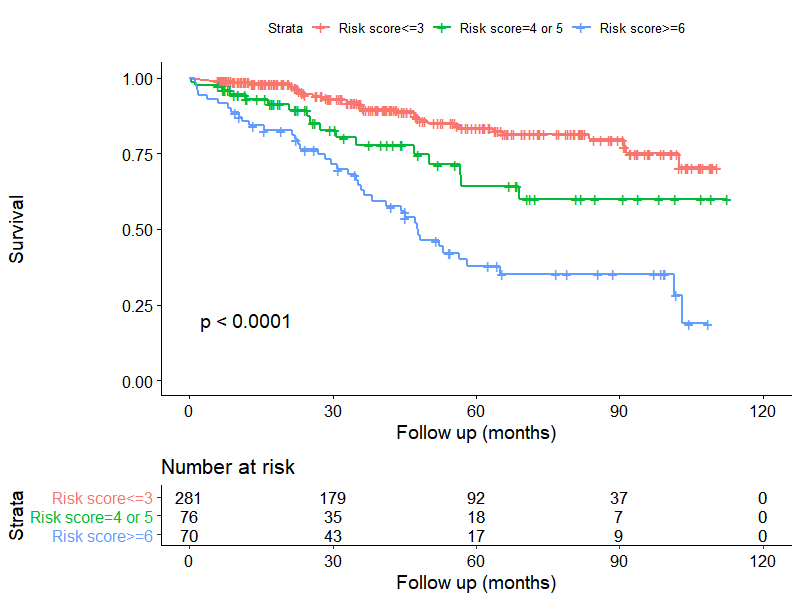
**

**Figure S5. Calibration of the derived model (A) and the risk score (B) in the validation cohort.**

**
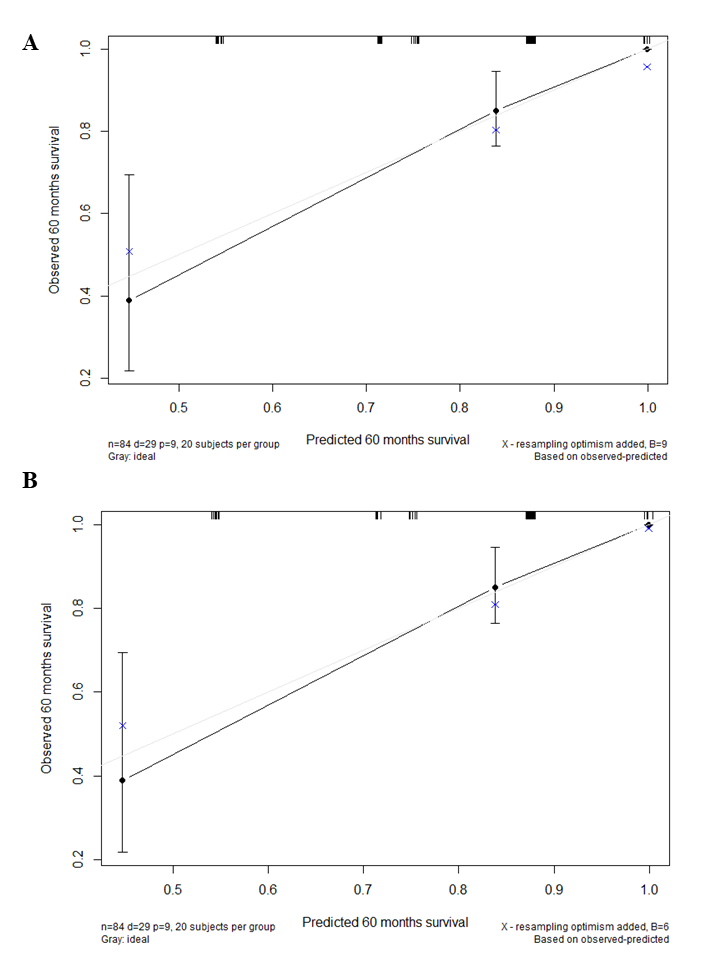
**

**Table S1. Univariate Cox proportional hazards analyses of candidate variables for all-cause mortality in the overall analyzed cohort.**

|  | Hazard ratio | 95% confidence interval | P value |
| --- | --- | --- | --- |
| Age/10 years | 1.154 | 0.968-1.376 | 0.111 |
| Females | 0.843 | 0.561-1.268 | 0.413 |
| BMI/5 kg·m^-2^ | 0.773 | 0.578-1.032 | 0.081 |
| Time from symptoms to diagnosis/12 months | 0.980 | 0.913-1.053 | 0.585 |
| Newly diagnosed | 0.782 | 0.513-1.191 | 0.251 |
| WHO-FC | | | |
| Ⅲ | 1.547 | 1.008-2.374 | **0.046** |
| Ⅳ | 2.033 | 0.982-4.206 | **0.006** |
| SBP/20 mmHg | 0.931 | 0.723-1.198 | 0.578 |
| DBP/20 mmHg | 0.975 | 0.808-1.177 | 0.792 |
| 6MWD/100 m | 0.768 | 0.639-0.924 | **0.005** |
| Borg dyspnea index/1 point | 1.057 | 0.960-1.163 | 0.260 |
| **Hemodynamics** | | | |
| HR/10 beats | 1.072 | 0.913-1.258 | 0.398 |
| SvO_2_/10% | 0.678 | 0.575-0.800 | **<0.001** |
| RAP/6 mmHg | 1.342 | 1.118-1.612 | **0.002** |
| mPAP/10 mmHg | 1.300 | 1.144-1.478 | **<0.001** |
| CI/1 L·min^-1^·m^-2^ | 0.510 | 0.358-0.725 | **<0.001** |
| PAWP/6 mmHg | 0.981 | 0.722-1.333 | 0.902 |
| PVR/300 dyn·s·cm^-5^ | 1.365 | 1.249-1.491 | **<0.001** |
| RVSP/10 mmHg | 1.156 | 1.059-1.261 | **0.001** |
| RVEDP/ 5 mmHg | 1.118 | 0.983-1.272 | 0.089 |
| **Laboratory test** | | | |
| NT-proBNP/500 fmol·L^-1^ | 1.069 | 1.036-1.104 | **<0.001** |
| Hemoglobin/1 g·L^-1^ | 1.044 | 0.940-1.160 | 0.420 |
| Uric acid/1 µmol·L^-1^ | 1.002 | 1.001-1.004 | **0.002** |
| Glucose/1 mmol·L^-1^ | 0.923 | 0.781-1.090 | 0.344 |
| TBiL/1 µmol·L^-1^ | 1.017 | 1.008-1.026 | **<0.001** |
| ALT/1 IU·L^-1^ | 1.003 | 0.994-1.012 | 0.499 |
| AST/1 IU·L^-1^ | 1.008 | 0.999-1.016 | 0.069 |
| Creatinine/10 µmol·L^-1^ | 1.147 | 1.039-1.266 | **0.007** |
| BUN/1 mmol·L^-1^ | 1.214 | 1.106-1.333 | **<0.001** |
| **Pulmonary function test** | | | |
| FEV1/10% predicted | 0.921 | 0.820-1.036 | 0.170 |
| FEV1/FVC /10% predicted | 0.999 | 0.994-1.004 | 0.692 |
| DLCO/10% predicted | 0.931 | 0.832-1.042 | 0.213 |
| **Echocardiography** | | | |
| LVEF/10 % | 0.949 | 0.743-1.212 | 0.673 |
| LAAPD/5 mm | 1.147 | 0.988-1.331 | 0.072 |
| LVEDD/5 mm | 0.927 | 0.807-1.063 | 0.278 |
| RVAPD/5 mm | 1.119 | 1.028-1.218 | **0.010** |
| **Comorbidities, n (%)** | | | |
| Atrial fibrillation | 1.677 | 0.680-4.134 | 0.261 |
| COPD | 1.285 | 0.315-5.236 | 0.726 |
| Coronary heart disease | 2.089 | 1.007-4.334 | **0.048** |
| Diabetes | 1.001 | 0.317-3.166 | 0.999 |
| Hypertension | 1.076 | 0.650-1.781 | 0.776 |
| Chronic kidney disease^*^ | 2.308 | 1.499-3.552 | **<0.001** |
| OSAS | 0.413 | 0.057-2.976 | 0.380 |
| Thyroid disease | 1.863 | 0.755-4.594 | 0.177 |
| Pulmonary embolism | 1.349 | 0.888-2.051 | 0.160 |
| Deep vein thrombosis | 0.794 | 0.463-1.361 | 0.401 |
| Obesity | 0.995 | 0.516-1.916 | 0.987 |
| Pericardial effusion | 1.295 | 0.705-2.377 | 0.404 |

* CKD stage 3 or more.

BMI: body mass index; WHO-FC: World Health Organization functional class; SBP: systolic blood pressure; DBP: diastolic blood pressure; 6MWD: 6 minute walking distance; SvO_2_: mixed venous oxygen saturation; HR: heart rate; RVSP: right ventricular systolic pressure; RVEDP: right ventricular end diastolic pressure; RAP: right atrial pressure; sPAP: systolic pulmonary artery pressure; dPAP: diastolic pulmonary artery pressure; mPAP: mean pulmonary artery pressure; CI: cardiac index; PAWP: pulmonary arterial wedge pressure; PVR: pulmonary vascular resistance; NT-proBNP: N-terminal pro b-type natriuretic peptide; TBIL: total bilirubin; ALT: alanine aminotransferase; AST: aspartate aminotransferase; BUN: blood urea nitrogen; FEV1: forced expiratory volume in 1 second; FVC: forced vital capacity; DLCO: diffusion capacity; LVEF: left ventricular ejection fraction; LAAPD: left atrial anteroposterior diameter; LVEDD: left ventricular end diastolic diameter; RVAPD: right ventricular anteroposterior diameter; COPD: chronic obstructive pulmonary disease; OSAS: obstructive sleep apnea syndrome.

**Table S2. Baseline characteristics of the 3 risk groups classified by the new derived risk score.**

|  | 0-3 points  N=285 | 4-5 points  N=76 | >= 6 points  N=71 | P value^#^ |
| --- | --- | --- | --- | --- |
| WHO function class, n (%) | | | | **<0.001** |
| Ⅰ/Ⅱ | 166 (58.2) | 27 (35.5) | 14 (19.7) |  |
| Ⅲ | 113 (39.6) | 43 (56.6) | 43 (60.6) |  |
| Ⅳ | 6 (2.1) | 6 (7.9) | 14 (19.7) |  |
| 6MWD (m) | 378.04 ± 99.34 | 313.16 ± 107.31 | 292.61 ± 104.03 | **<0.001** |
| Borg dyspnea index | 2.49 ± 1.90 | 2.82 ± 2.17 | 3.75 ± 1.99 | **<0.001** |
| **Hemodynamics** | | | | |
| SvO_2_(%) | 66.12 ± 9.41 | 61.63 ± 9.36 | 56.15 ± 12.33 | **<0.001** |
| HR (beats) | 81.12 ± 13.48 | 80.86 ± 14.71 | 84.73 ± 13.29 | 0.145 |
| RVSP (mmHg) | 82.52 ± 22.67 | 89.72 ± 21.74 | 100.83 ± 22.79 | **<0.001** |
| RVEDP (mmHg) | 7.94 ± 7.21 | 10.47 ± 8.43 | 12.13 ± 8.45 | **<0.001** |
| RAP (mmHg) | 5.79 ± 4.49 | 8.53 ± 5.80 | 11.34 ± 6.42 | **<0.001** |
| sPAP (mmHg) | 83.08 ± 19.96 | 89.61 ± 17.58 | 101.30 ± 22.65 | **<0.001** |
| dPAP (mmHg) | 29.24 ± 9.91 | 32.32 ± 9.38 | 39.90 ± 14.97 | **<0.001** |
| mPAP (mmHg) | 47.29 ± 11.52 | 51.92 ± 10.22 | 61.03 ± 17.19 | **<0.001** |
| CI (L·min^-1^·m^-2^) | 2.55 ± 0.87 | 2.17 ± 0.57 | 1.76 ± 0.54 | **<0.001** |
| PAWP (mm Hg) | 7.82 ± 3.31 | 9.05 ± 3.45 | 8.58 ± 3.24 | **0.011** |
| PVR (dyn·s·cm^-5^) | 834.41 ± 343.42 | 1018.47 ± 303.5 | 1732.44 ± 659.79 | **<0.001** |
| **Laboratory test** | | | | |
| NT-proBNP (fmol/L) ^*^ | 428.30 (120.17, 1522.50) | 1582.50 (550.46, 3099.00) | 2035.00 (559.70, 4420.00) | **<0.001** |
| TBIL (µmol/L) | 17.86 ± 8.09 | 30.35 ± 19.17 | 32.33 ± 20.97 | **<0.001** |
| **Comorbidities, n (%)** | | | | |
| Chronic kidney disease^**^ | 8 (2.8) | 54 (71.7) | 30 (42.3) | **<0.001** |
| The Swedish/COMPERA risk stratum | | | | |
| Low risk | 98 (34.4) | 0 | 0 |  |
| Intermediate risk | 187 (65.6) | 76 (100%) | 53 (74.6) |  |
| High risk | 0 | 0 | 18 (25.4) |  |

^#^ comparison between 3 risk groups; ^*^ median (interquartile range); ^**^ CKD stage 3 or more.

WHO-FC: World Health Organization functional class; 6MWD: 6 minute walking distance; SvO_2_: mixed venous oxygen saturation; HR: heart rate; RVSP: right ventricular systolic pressure; RVEDP: right ventricular end diastolic pressure; RAP: right atrial pressure; sPAP: systolic pulmonary artery pressure; dPAP: diastolic pulmonary artery pressure; mPAP: mean pulmonary artery pressure; CI: cardiac index; PAWP: pulmonary arterial wedge pressure; PVR: pulmonary vascular resistance; NT-proBNP: N-terminal pro b-type natriuretic peptide; TBIL: total bilirubin.

**Table S3. Baseline characteristics of the newly diagnosed CTEPH patients.**

|  | All  N=256 | Survivors  N=197 | Non-survivors  N=59 | P value^#^ |
| --- | --- | --- | --- | --- |
| Age (years) | 54.72 ± 12.1 | 54.35 ± 12.09 | 55.97 ± 12.13 | 0.308 |
| Males, n (%) | 137 (53.5) | 103 (52.3) | 34 (57.6) | 0.470 |
| BMI (kg/m^2^) | 24.59 ± 4.00 | 24.75 ± 3.83 | 24.06 ± 4.51 | 0.098 |
| Time from symptoms to diagnosis (months) | 35.76 ± 33.37 | 35.11 ± 32.44 | 37.93 ± 36.52 | 0.558 |
| WHO function class, n (%) | | | | 0.168 |
| Ⅰ/Ⅱ | 141 (55.1) | 112 (56.9) | 29 (49.2) |  |
| Ⅲ | 101 (39.5) | 76 (38.6) | 25 (42.4) |  |
| Ⅳ | 14 (5.5) | 9 (4.6) | 5 (8.5) |  |
| SBP | 118.8 ± 17.34 | 119.26 ± 17.26 | 117.24 ± 17.7 | 0.445 |
| DBP | 77.51 ± 11.35 | 77.37 ± 11.39 | 77.98 ± 11.29 | 0.495 |
| 6MWD (m) | 350.73 ± 111.22 | 356.42 ± 109 | 331.71 ± 117.32 | 0.229 |
| Borg dyspnea index | 2.67 ± 2.08 | 2.58 ± 2.07 | 2.95 ± 2.09 | 0.261 |
| **Hemodynamics** | | | | |
| SvO_2_(%) | 63.29 ± 11.69 | 64.36 ± 11.1 | 59.73 ± 12.96 | 0.019 |
| HR (beats) | 82.18 ± 14.27 | 82.36 ± 14.4 | 81.56 ± 13.94 | 0.444 |
| RVSP (mmHg) | 84.96 ± 23.73 | 82.86 ± 23.91 | 92 ± 21.88 | 0.007 |
| RVEDP (mmHg) | 9.17 ± 7.45 | 8.84 ± 7.42 | 10.25 ± 7.53 | 0.229 |
| RAP (mmHg) | 7.1 ± 5.64 | 6.5 ± 4.94 | 9.08 ± 7.24 | 0.035 |
| sPAP (mmHg) | 85.99 ± 20.75 | 84.13 ± 20.37 | 92.19 ± 20.99 | 0.008 |
| dPAP (mmHg) | 30.51 ± 10.88 | 29.52 ± 9.57 | 33.83 ± 14.02 | 0.020 |
| mPAP (mmHg) | 49.5 ± 12.67 | 48.03 ± 11.34 | 54.42 ± 15.45 | 0.005 |
| CI (L·min^-1^·m^-2^) | 2.2 ± 0.8 | 2.31 ± 0.83 | 1.84 ± 0.55 | <0.001 |
| PAWP (mm Hg) | 8.03 ± 3.17 | 8.01 ± 3.38 | 8.08 ± 2.37 | 0.890 |
| PVR (dyn·s·cm^-5^) | 1022.36 ± 538.61 | 914.88 ± 431.03 | 1381.27 ± 690.9 | <0.001 |
| **Laboratory test** | | | | |
| NT-proBNP (fmol/L) ^*^ | 908.90(260.78,2458.00) | 726.90 (211.40,2202.00) | 1715.00 (540.80,4256.00) | 0.001 |
| Hemoglobin | 146.37 ± 20.26 | 146.51 ± 20.24 | 145.91 ± 20.51 | 0.714 |
| Uric acid (µmol/L) | 402.98 ± 117.45 | 403.44 ± 121.74 | 401.47 ± 102.77 | 0.951 |
| Glucose (mmol/L) | 5.31 ± 1.39 | 5.31 ± 1.27 | 5.3 ± 1.74 | 0.367 |
| TBiL (µmol/L) | 20.49 ± 15.01 | 19.56 ± 14.59 | 23.6 ± 16.09 | 0.051 |
| ALT (IU/L) | 28.65 ± 20.35 | 27.47 ± 18.68 | 32.58 ± 24.91 | 0.542 |
| AST (IU/L) | 29.42 ± 21.82 | 28.38 ± 19.94 | 32.87 ± 27.1 | 0.085 |
| Creatinine (µmol/L) | 81.48 ± 18.57 | 79.98 ± 18.7 | 86.5 ± 17.37 | 0.013 |
| BUN (mmol/L) | 6.25 ± 1.92 | 6.1 ± 1.78 | 6.78 ± 2.24 | 0.020 |
| **Pulmonary function test** | | | | |
| FEV1 (% predicted) | 82.96 ± 16.6 | 83.64 ± 16.5 | 80.67 ± 16.86 | 0.148 |
| FEV1/FVC (% predicted) | 44.33 ± 38.55 | 46.01 ± 38.69 | 38.73 ± 37.88 | 0.158 |
| DLCO (% predicted) | 67.69 ± 18.21 | 68.15 ± 18.9 | 66.16 ± 15.74 | 0.263 |
| **Echocardiography** | | | | |
| LVEF (%) | 63.96 ± 7.85 | 63.87 ± 7.82 | 64.28 ± 8.01 | 0.766 |
| LAAPD (mm) | 32.73 ± 6.56 | 32.73 ± 6.4 | 32.72 ± 7.11 | 0.812 |
| LVEDD (mm) | 36.73 ± 7.69 | 37.15 ± 7.67 | 35.33 ± 7.68 | 0.085 |
| RVAPD (mm) | 40.49 ± 12.04 | 39.16 ± 12.3 | 44.95 ± 10.01 | 0.001 |
| **Comorbidities, n (%)** | | | | |
| Atrial fibrillation | 14 (5.5) | 5 (2.5) | 3 (5.1) | 0.324 |
| COPD | 6 (2.3) | 5 (2.5) | 1 (1.7) | 0.707 |
| Coronary heart disease | 15 (5.9) | 13 (6.6) | 2 (3.4) | 0.357 |
| Diabetes | 4 (1.6) | 2 (1.0) | 2 (3.4) | 0.197 |
| Hypertension | 54 (21.1) | 42 (21.3) | 12 (20.3) | 0.871 |
| Chronic kidney disease^**^ | 49 (19.1) | 33 (16.8) | 16 (27.1) | 0.076 |
| OSAS | 4 (1.6) | 4 (2.0) | 0 | 0.270 |
| Thyroid disease | 6 (2.3) | 5 (2.5) | 1 (1.7) | 0.707 |
| Pulmonary embolism | 137 (53.5) | 102 (51.8) | 35 (59.3) | 0.308 |
| Deep vein thrombosis | 53 (20.7) | 46 (23.4) | 7 (11.9) | 0.056 |
| Obesity | 37 (14.5) | 30 (15.2) | 7 (11.9) | 0.519 |
| Pericardial effusion | 14 (5.5) | 10 (5.1) | 4 (6.8) | 0.614 |
| Targeted drugs, n (%) | 115 (44.9) | 89 (45.2) | 26 (44.1) | 0.880 |
| Combination therapy, n (%) | 17 (6.6) | 13 (6.6) | 4 (6.8) | 0.947 |

^#^ comparison between survivors and non-survivors; ^*^ median (interquartile range); ^**^ CKD stage 3 or more.

BMI: body mass index; WHO-FC: World Health Organization functional class; SBP: systolic blood pressure; DBP: diastolic blood pressure; 6MWD: 6 minute walking distance; SvO_2_: mixed venous oxygen saturation; HR: heart rate; RVSP: right ventricular systolic pressure; RVEDP: right ventricular end diastolic pressure; RAP: right atrial pressure; sPAP: systolic pulmonary artery pressure; dPAP: diastolic pulmonary artery pressure; mPAP: mean pulmonary artery pressure; CI: cardiac index; PAWP: pulmonary arterial wedge pressure; PVR: pulmonary vascular resistance; NT-proBNP: N-terminal pro b-type natriuretic peptide; TBIL: total bilirubin; ALT: alanine aminotransferase; AST: aspartate aminotransferase; BUN: blood urea nitrogen; FEV1: forced expiratory volume in 1 second; FVC: forced vital capacity; DLCO: diffusion capacity; LVEF: left ventricular ejection fraction; LAAPD: left atrial anteroposterior diameter; LVEDD: left ventricular end diastolic diameter; RVAPD: right ventricular anteroposterior diameter; COPD: chronic obstructive pulmonary disease; OSAS: obstructive sleep apnea syndrome.

**Table S4. Baseline characteristics of the surgically inoperable CTEPH patients.**

|  | All  N=234 | Survivors  N=189 | Non-survivors  N=45 | P value^#^ |
| --- | --- | --- | --- | --- |
| Age (years) | 53.96 ± 12.23 | 53.46 ± 12.33 | 56.07 ± 11.7 | 0.190 |
| Males, n (%) | 111 (47.4) | 88 (46.6) | 23 (51.1) | 0.583 |
| BMI (kg/m^2^) | 24.13 ± 3.99 | 24.33 ± 3.82 | 23.28 ± 4.57 | 0.032 |
| Time from symptoms to diagnosis (months) | 27.22 ± 29.51 | 27.2 ± 29.6 | 27.34 ± 29.44 | 0.753 |
| Incident, n (%) | 154 (65.8) | 122 (64.6) | 32 (71.1) | 0.404 |
| WHO function class, n (%) | | | | 0.087 |
| Ⅰ/Ⅱ | 121 (51.7) | 101 (53.5) | 20 (44.4) |  |
| Ⅲ | 102 (43.6) | 82 (43.4) | 20 (44.4) |  |
| Ⅳ | 11 (4.7) | 6 (3.2) | 5 (11.1) |  |
| SBP | 117.84 ± 16.57 | 118.57 ± 16.33 | 114.78 ± 17.41 | 0.180 |
| DBP | 76.74 ± 11.27 | 76.79 ± 11.3 | 76.49 ± 11.23 | 0.836 |
| 6MWD (m) | 357.85 ± 111.04 | 367.03 ± 105.6 | 319.33 ± 125.58 | 0.055 |
| Borg dyspnea index | 2.65 ± 1.96 | 2.59 ± 1.94 | 2.89 ± 2.08 | 0.503 |
| **Hemodynamics** | | | | |
| SvO_2_(%) | 63.46 ± 10.64 | 64.53 ± 10.29 | 58.95 ± 11.02 | 0.002 |
| HR (beats) | 81.73 ± 14.04 | 81.89 ± 14.57 | 81.07 ± 11.72 | 0.743 |
| RVSP (mmHg) | 83.95 ± 23.96 | 81.96 ± 23.95 | 92.29 ± 22.39 | 0.013 |
| RVEDP (mmHg) | 8.72 ± 7.34 | 8.28 ± 7.45 | 10.58 ± 6.63 | 0.021 |
| RAP (mmHg) | 7.18 ± 5.63 | 6.59 ± 5.13 | 9.67 ± 6.89 | 0.004 |
| sPAP (mmHg) | 85.61 ± 20.49 | 83.72 ± 20.03 | 93.53 ± 20.76 | 0.007 |
| dPAP (mmHg) | 30.94 ± 11.66 | 29.98 ± 10.24 | 34.98 ± 15.84 | 0.022 |
| mPAP (mmHg) | 49.33 ± 13.05 | 47.97 ± 11.8 | 55.02 ± 16.31 | 0.009 |
| CI (L·min^-1^·m^-2^) | 2.4 ± 0.9 | 2.52 ± 0.92 | 1.89 ± 0.61 | <0.001 |
| PAWP (mm Hg) | 8.17 ± 3.24 | 8.08 ± 3.32 | 8.53 ± 2.9 | 0.419 |
| PVR (dyn·s·cm^-5^) | 991.94 ± 564.21 | 890.9 ± 424.36 | 1416.29 ± 829.62 | <0.001 |
| **Laboratory test** | | | | |
| NT-proBNP (fmol/L) ^*^ | 769.19 (181.51,2132.50) | 650.70 (136.95,1852.00) | 1889.00 (637.34,4453.50) | <0.001 |
| Hemoglobin | 145.94 ± 21.11 | 146.68 ± 20.94 | 142.86 ± 21.77 | 0.374 |
| Uric acid (µmol/L) | 410.68 ± 120.71 | 405.59 ± 121.51 | 432.04 ± 116.19 | 0.200 |
| Glucose (mmol/L) | 5.24 ± 1.25 | 5.29 ± 1.26 | 5.06 ± 1.2 | 0.261 |
| TBiL (µmol/L) | 21.16 ± 12.55 | 20.19 ± 11.43 | 25.23 ± 15.97 | 0.033 |
| ALT (IU/L) | 29.36 ± 22.52 | 29.24 ± 22.16 | 29.87 ± 24.24 | 0.547 |
| AST (IU/L) | 29.6 ± 23.38 | 28.77 ± 21.95 | 33.09 ± 28.67 | 0.098 |
| Creatinine (µmol/L) | 80.39 ± 19.83 | 78.56 ± 19.61 | 88.08 ± 19.07 | 0.003 |
| BUN (mmol/L) | 6.4 ± 1.91 | 6.14 ± 1.81 | 7.49 ± 1.96 | <0.001 |
| **Pulmonary function test** | | | | |
| FEV1 (% predicted) | 82.33 ± 17.17 | 82.56 ± 17.16 | 81.36 ± 17.35 | 0.568 |
| FEV1/FVC (% predicted) | 48.00 ± 41.20 | 51.68 ± 41.24 | 32.57 ± 37.68 | 0.001 |
| DLCO (% predicted) | 68.16 ± 17.33 | 68.74 ± 16.85 | 65.73 ± 19.21 | 0.262 |
| **Echocardiography** | | | | |
| LVEF (%) | 64.01 ± 7.15 | 63.84 ± 6.63 | 64.72 ± 9.05 | 0.499 |
| LAAPD (mm) | 32.17 ± 5.88 | 32.34 ± 6.1 | 31.49 ± 4.85 | 0.558 |
| LVEDD (mm) | 37.86 ± 7.03 | 38.07 ± 7.08 | 36.99 ± 6.83 | 0.206 |
| RVAPD (mm) | 38.61 ± 11.23 | 37.44 ± 11.38 | 43.51 ± 9.14 | <0.001 |
| **Comorbidities, n (%)** | | | | |
| Atrial fibrillation | 8 (3.4) | 7 (3.7) | 1 (2.2) | 0.623 |
| COPD | 5 (2.1) | 4 (2.1) | 1 (2.2) | 0.965 |
| Coronary heart disease | 14 (6.0) | 12 (8.3) | 2 (4.4) | 0.628 |
| Diabetes | 13 (5.6) | 12 (6.3) | 1 (2.2) | 0.277 |
| Hypertension | 46 (19.7) | 39 (20.6) | 7 (15.6) | 0.441 |
| Chronic kidney disease^**^ | 49 (20.9) | 29 (15.3) | 20 (44.4) | <0.001 |
| OSAS | 14 (6.0) | 14 (7.4) | 0 | 0.060 |
| Thyroid disease | 9 (3.8) | 6 (3.2) | 3 (6.7) | 0.274 |
| Pulmonary embolism | 134 (57.3) | 110 (58.2) | 24 (53.3) | 0.553 |
| Deep vein thrombosis | 49 (20.9) | 41 (21.7) | 8 (17.8) | 0.562 |
| Obesity | 29 (12.4) | 24 (12.7) | 5 (11.1) | 0.771 |
| Pericardial effusion | 20 () | 12 | 8 | 0.014 |
| Targeted drugs, n (%) | 133 (56.8) | 108 (57.1) | 25 (55.6) | 0.847 |
| Combination therapy, n (%) | 16 (6.8) | 14 (7.4) | 2 (4.4) | 0.724 |

^#^ comparison between survivors and non-survivors; ^*^ median (interquartile range); ^**^ CKD stage 3 or more.

BMI: body mass index; WHO-FC: World Health Organization functional class; SBP: systolic blood pressure; DBP: diastolic blood pressure; 6MWD: 6 minute walking distance; SvO_2_: mixed venous oxygen saturation; HR: heart rate; RVSP: right ventricular systolic pressure; RVEDP: right ventricular end diastolic pressure; RAP: right atrial pressure; sPAP: systolic pulmonary artery pressure; dPAP: diastolic pulmonary artery pressure; mPAP: mean pulmonary artery pressure; CI: cardiac index; PAWP: pulmonary arterial wedge pressure; PVR: pulmonary vascular resistance; NT-proBNP: N-terminal pro b-type natriuretic peptide; TBIL: total bilirubin; ALT: alanine aminotransferase; AST: aspartate aminotransferase; BUN: blood urea nitrogen; FEV1: forced expiratory volume in 1 second; FVC: forced vital capacity; DLCO: diffusion capacity; LVEF: left ventricular ejection fraction; LAAPD: left atrial anteroposterior diameter; LVEDD: left ventricular end diastolic diameter; RVAPD: right ventricular anteroposterior diameter; COPD: chronic obstructive pulmonary disease; OSAS: obstructive sleep apnea syndrome.

**Table S5. Baseline characteristics of patients without chronic liver disease.**

|  | All  N=427 | Survivors  N=334 | Non-survivors  N=93 | P value^#^ |
| --- | --- | --- | --- | --- |
| Age (years) | 53.58 ± 12.27 | 53.29 ± 12.26 | 54.61 ± 12.31 | 0.230 |
| Males, n (%) | 225 (52.7) | 172 (51.5) | 53 (57.0) | 0.348 |
| BMI (kg/m^2^) | 24.00 ± 3.90 | 24.14 ± 3.81 | 23.51 ± 4.17 | 0.085 |
| Time from symptoms to diagnosis (months) | 29.65 ± 33.35 | 29.19 ± 32.71 | 31.29 ± 35.69 | 0.399 |
| Incident, n (%) | 252 (59.0) | 194 (58.1) | 58 (62.4) | 0.458 |
| WHO function class, n (%) | | | | 0.083 |
| Ⅰ/Ⅱ | 205 (48.0) | 167 (50.0) | 38 (40.9) |  |
| Ⅲ | 196 (45.9) | 150 (44.9) | 46 (49.5) |  |
| Ⅳ | 26 (6.0) | 17 (5.1) | 9 (9.7) |  |
| SBP | 116.99 ± 16.85 | 117.34 ± 16.71 | 115.74 ± 17.39 | 0.429 |
| DBP | 78.78 ± 40.22 | 79.17 ± 45.1 | 77.35 ± 11.17 | 0.393 |
| 6MWD (m) | 353.5 ± 107.38 | 362.08 ± 102.81 | 322.67 ± 117.92 | 0.009 |
| Borg dyspnea index | 2.77 ± 2.01 | 2.71 ± 1.98 | 3.01 ± 2.09 | 0.338 |
| **Hemodynamics** | | | | |
| SvO_2_(%) | 63.66 ± 10.61 | 64.89 ± 9.96 | 59.27 ± 11.7 | <0.001 |
| HR (beats) | 81.6 ± 13.70 | 81.39 ± 13.88 | 82.38 ± 13.07 | 0.605 |
| RVSP (mmHg) | 86.76 ± 23.59 | 85.11 ± 23.75 | 92.71 ± 22.09 | 0.008 |
| RVEDP (mmHg) | 9.06 ± 7.83 | 8.46 ± 7.79 | 11.2 ± 7.6 | 0.001 |
| RAP (mmHg) | 7.2 ± 5.51 | 6.61 ± 4.96 | 9.34 ± 6.77 | 0.001 |
| sPAP (mmHg) | 87.23 ± 21.18 | 85.57 ± 20.82 | 93.17 ± 21.52 | 0.004 |
| dPAP (mmHg) | 31.55 ± 11.53 | 30.68 ± 10.7 | 34.66 ± 13.74 | 0.007 |
| mPAP (mmHg) | 50.37 ± 13.44 | 49.11 ± 12.56 | 54.88 ± 15.49 | 0.001 |
| CI (L·min^-1^·m^-2^) | 2.36 ± 0.84 | 2.48 ± 0.86 | 1.92 ± 0.57 | <0.001 |
| PAWP (mm Hg) | 8.16 ± 3.36 | 8.17 ± 3.5 | 8.11 ± 2.79 | 0.885 |
| PVR (dyn·s·cm^-5^) | 1010.26 ± 518.51 | 912.38 ± 419.32 | 1361.81 ± 669.89 | <0.001 |
| **Laboratory test** | | | | |
| NT-proBNP (fmol/L) ^*^ | 731.30 (183.25, 2202.00) | 650.7 (163.53, 2097.5) | 1401 (347.04-3730.00) | 0.002 |
| Hemoglobin | 149.73 ± 20.38 | 149.81 ± 19.99 | 149.42 ± 21.83 | 0.719 |
| Uric acid (µmol/L) | 419.57 ± 126.27 | 413.12 ± 121.26 | 442.76 ± 141.12 | 0.101 |
| Glucose (mmol/L) | 5.23 ± 1.26 | 5.27 ± 1.18 | 5.07 ± 1.49 | 0.019 |
| TBiL (µmol/L) | 22.57 ± 14.84 | 21.17 ± 14.05 | 27.57 ± 16.54 | <0.001 |
| ALT (IU/L) | 29.03 ± 21.49 | 28.74 ± 21.37 | 30.1 ± 21.98 | 0.788 |
| AST (IU/L) | 28.63 ± 19.57 | 27.9 ± 18.63 | 31.25 ± 22.53 | 0.017 |
| Creatinine (µmol/L) | 82.39 ± 18.98 | 81.05 ± 18.88 | 87.23 ± 18.67 | 0.006 |
| BUN (mmol/L) | 6.4 ± 2.01 | 6.2 ± 1.88 | 7.09 ± 2.28 | <0.001 |
| **Pulmonary function test** | | | | |
| FEV1 (% predicted) | 80.4 ± 16.89 | 80.93 ± 16.81 | 78.52 ± 17.1 | 0.160 |
| DLCO (% predicted) | 67.4 ± 17.39 | 67.95 ± 17.5 | 65.45 ± 16.94 | 0.121 |
| **Echocardiography** | | | | |
| LVEF (%) | 64.07 ± 7.49 | 63.98 ± 7.12 | 64.41 ± 8.73 | 0.771 |
| LAAPD (mm) | 32.7 ± 6.14 | 32.58 ± 5.95 | 33.12 ± 6.78 | 0.734 |
| LVEDD (mm) | 37.51 ± 7.13 | 37.97 ± 6.99 | 35.88 ± 7.42 | 0.009 |
| RVAPD (mm) | 38.83 ± 11.11 | 37.6 ± 11.21 | 43.26 ± 9.56 | <0.001 |
| **Comorbidities, n (%)** | | | | |
| Atrial fibrillation | 17 (3.9) | 12 (3.6) | 5 (5.4) | 0.437 |
| COPD | 10 (2.3) | 8 (2.4) | 2 (2.2) | 0.890 |
| Coronary heart disease | 31 (7.2) | 23 (6.9) | 8 (8.6) | 0.573 |
| Diabetes | 18 (4.2) | 15 (4.5) | 3 (3.2) | 0.591 |
| Hypertension | 88 (20.6) | 69 (20.7) | 19 (20.4) | 0.963 |
| Chronic kidney disease^**^ | 92 (21.3) | 61 (18.3) | 31 (33.3) | 0.002 |
| OSAS | 27 (6.3) | 26 (7.8) | 1 (1.1) | 0.019 |
| Thyroid disease | 14 (3.2) | 9 (2.7) | 5 (5.4) | 0.199 |
| Pulmonary embolism | 248 (58.1) | 190 (56.9) | 58 (62.4) | 0.344 |
| Deep vein thrombosis | 102 (23.9) | 87 (26.0) | 15 (16.1) | 0.047 |
| Obesity | 46 (10.8) | 36 (10.8) | 10 (10.8) | 0.994 |
| Pericardial effusion | 41 (9.6) | 29 (8.7) | 12 (12.9) | 0.222 |
| Targeted drugs, n (%) | 238 (55.7) | 192 (57.5) | 46 (49.5) | 0.168 |
| Combination therapy, n (%) | 40 (9.4) | 34 (10.2) | 6 (6.5) | 0.190 |

^#^ comparison between survivors and non-survivors; ^*^ median (interquartile range); ^**^ CKD stage 3 or more.

BMI: body mass index; WHO-FC: World Health Organization functional class; SBP: systolic blood pressure; DBP: diastolic blood pressure; 6MWD: 6 minute walking distance; SvO_2_: mixed venous oxygen saturation; HR: heart rate; RVSP: right ventricular systolic pressure; RVEDP: right ventricular end diastolic pressure; RAP: right atrial pressure; sPAP: systolic pulmonary artery pressure; dPAP: diastolic pulmonary artery pressure; mPAP: mean pulmonary artery pressure; CI: cardiac index; PAWP: pulmonary arterial wedge pressure; PVR: pulmonary vascular resistance; NT-proBNP: N-terminal pro b-type natriuretic peptide; TBIL: total bilirubin; ALT: alanine aminotransferase; AST: aspartate aminotransferase; BUN: blood urea nitrogen; FEV1: forced expiratory volume in 1 second; DLCO: diffusion capacity; LVEF: left ventricular ejection fraction; LAAPD: left atrial anteroposterior diameter; LVEDD: left ventricular end diastolic diameter; RVAPD: right ventricular anteroposterior diameter; COPD: chronic obstructive pulmonary disease; OSAS: obstructive sleep apnea syndrome

**Table S6. Estimated 1-, 3- and 5-year survival of the three risk groups in newly diagnosed CTEPH patients.**

| Risk score | number | Estimated 1-year survival，% (95% CI) | Estimated 3-year survival，% (95% CI) | Estimated 5-year survival，% (95% CI) |
| --- | --- | --- | --- | --- |
| 0-3 | 171 | 98.2 (96.3-1.00) | 91.2 (86.8-95.9) | 85.6 (79.7-92.0) |
| 4-5 | 43 | 95.3 (89.3-1.00) | 83.2 (71.6-96.7) | 68.1 (51.8-89.5) |
| >=6 | 42 | 85.7 (75.8-1.00) | 63.0 (49.7-79.9) | 34.4 (21.3-55.3) |

CI: confidence interval.

**Table S7. Estimated 1-, 3- and 5-year survival of the three risk groups in surgically inoperable patients.**

| Risk score | number | Estimated 1-year survival，% (95% CI) | Estimated 3-year survival，% (95% CI) | Estimated 5-year survival，% (95% CI) |
| --- | --- | --- | --- | --- |
| 0-3 | 160 | 99.4 (98.2-1.00) | 95.1 (91.4-99.1) | 88.1 (81.3-95.3) |
| 4-5 | 41 | 95.1 (88.6-1.00) | 78.3 (63.8-96.0) | 55.5 (37.3-82.6) |
| >=6 | 33 | 78.8 (66.0-94.0) | 59.4 (44.5-79.3) | 44.1 (29.2-66.6) |

CI: confidence interval.

**Table S8. Estimated 1-, 3- and 5-year survival of the three risk groups in patients without chronic liver disease.**

| Risk score | number | Estimated 1-year survival，% (95% CI) | Estimated 3-year survival，% (95% CI) | Estimated 5-year survival，% (95% CI) |
| --- | --- | --- | --- | --- |
| 0-3 | 281 | 98.5 (97.1-1) | 91.3 (87.6-95.2) | 83.4 (78.0-89.3) |
| 4-5 | 76 | 93.0 (87.2-99.1) | 77.8 (67.0-90.3) | 64.3 (50.6-81.7) |
| >=6 | 70 | 85.6 (77.8-94.3) | 62.8 (51.9-75.9) | 37.7 (26.8-53.1) |

CI: confidence interval.

**Table S9. Baseline characteristics of the validation cohort. ^#^**

|  | All  N=84 |
| --- | --- |
| Age (years) | 52.01 ± 52.01 |
| Males, n (%) | 50 (59.5) |
| BMI (kg/m^2^) | 23.75 ± 23.75 |
| Time from symptoms to diagnosis (months) | 33.32 ± 48.79 |
| Newly diagnosed, n (%) | 30 (35.7) |
| WHO-FC, n (%) | |
| Ⅰ/Ⅱ | 40 (47.6) |
| Ⅲ | 39 (46.4) |
| Ⅳ | 5 (6.0) |
| SBP | 118.75 ± 14.61 |
| DBP | 75.61 ± 10.90 |
| 6MWD (m) | 332.06 ± 332.06 |
| Borg dyspnea index | 2.76 ± 2.76 |
| **Hemodynamics** | |
| SvO_2_(%) | 61.45 ± 61.45 |
| HR (beats) | 80.71 ± 80.71 |
| RVSP (mmHg) | 85.33 ± 85.33 |
| RVEDP (mmHg) | 9.30 ± 6.98 |
| RAP (mmHg) | 9.73 ± 9.73 |
| sPAP (mmHg) | 84.62 ± 84.62 |
| dPAP (mmHg) | 30.93 ± 30.93 |
| mPAP (mmHg) | 49.65 ± 49.65 |
| CI (L·min^-1^·m^-2^) | 1.98 ± 1.98 |
| PAWP (mm Hg) | 9.21 ± 9.21 |
| PVR (dyn·s·cm^-5^) | 1100.2 ± 1100.2 |
| **Laboratory results** | |
| NT-proBNP (fmol/L) ^*^ | 646.50 (240.34,1529.50) |
| Hemoglobin | 146.87 ± 21.28 |
| Uric acid (µmol/L) | 454.01 ± 454.01 |
| Glucose (mmol/L) | 4.63 ± 0.95 |
| TBIL (µmol/L) | 24.48 ± 24.48 |
| ALT (IU/L) | 31.32 ± 31.32 |
| AST (IU/L) | 29.14 ± 29.14 |
| Creatinine (µmol/L) | 83.16 ± 83.16 |
| BUN (mmol/L) | 6.03 ± 6.03 |
| **Pulmonary function test** | |
| FEV1 (% predicted) | 81.32 ± 81.32 |
| FEV1/FVC (% predicted) | 83.39 ± 23.90 |
| DLCO (% predicted) | 68.56 ± 68.56 |
| **Echocardiography** | |
| LVEF (%) | 66.48 ± 66.48 |
| LAAPD (mm) | 31.52 ± 31.52 |
| LVEDD (mm) | 36.63 ± 36.63 |
| RVAPD (mm) | 39.86 ± 39.86 |
| **Comorbidities, n (%)** | |
| Atrial fibrillation | 6 (7.1) |
| COPD | 1 (1.2) |
| Coronary heart disease | 1 (1.2) |
| Diabetes | - |
| Hypertension | 14 (16.7) |
| Chronic kidney disease^**^ | 14 (16.7) |
| OSAS | - |
| Thyroid disease | 6 (7.1) |
| Pulmonary embolism | 48 (57.1) |
| Deep vein thrombosis | 19 (22.6) |
| Obesity | 10 (11.9) |
| Pericardial effusion | 8 (9.5) |
| Targeted drugs, n (%) | 25 (29.8) |
| Combination therapy, n (%) | 22 (26.2) |

^#^ Comparisons between survivors and non-survivors were not performed due to the relatively small sample size of subgroups; ^*^ median (interquartile range); ^**^ CKD stage 3 or more.

BMI: body mass index; WHO-FC: World Health Organization functional class; SBP: systolic blood pressure; DBP: diastolic blood pressure; 6MWD: 6 minute walking distance; SvO_2_: mixed venous oxygen saturation; HR: heart rate; RVSP: right ventricular systolic pressure; RVEDP: right ventricular end diastolic pressure; RAP: right atrial pressure; sPAP: systolic pulmonary artery pressure; dPAP: diastolic pulmonary artery pressure; mPAP: mean pulmonary artery pressure; CI: cardiac index; PAWP: pulmonary arterial wedge pressure; PVR: pulmonary vascular resistance; NT-proBNP: N-terminal pro b-type natriuretic peptide; TBIL: total bilirubin; ALT: alanine aminotransferase; AST: aspartate aminotransferase; BUN: blood urea nitrogen; FEV1: forced expiratory volume in 1 second; FVC: forced vital capacity; DLCO: diffusion capacity; LVEF: left ventricular ejection fraction; LAAPD: left atrial anteroposterior diameter; LVEDD: left ventricular end diastolic diameter; RVAPD: right ventricular anteroposterior diameter; COPD: chronic obstructive pulmonary disease; OSAS: obstructive sleep apnea syndrome.
